# An Anticancer Role of Hydrogen Sulfide in Human Gastric Cancer Cells

**DOI:** 10.1155/2015/636410

**Published:** 2015-05-11

**Authors:** Li Zhang, Qi Qi, Jianqiang Yang, Dongsheng Sun, Chunfeng Li, Yingwei Xue, Qiuying Jiang, Ye Tian, Changqing Xu, Rui Wang

**Affiliations:** ^1^Department of Pathophysiology, Harbin Medical University, Harbin 150081, China; ^2^Department of Pathology, the First Affiliated Hospital of Heilongjiang University of Chinese Medicine, Harbin 150040, China; ^3^Department of Pathophysiology, Qiqihar Medical College, Qiqihar 161006, China; ^4^Department of Gastrointestinal Surgery, the Third Affiliated Hospital, Harbin Medical University, Harbin 150081, China; ^5^Department of Oncology, the Second Affiliated Hospital, Harbin Medical University, Harbin 150081, China; ^6^Department of Biology, Lakehead University, Thunder Bay, ON, Canada P7B 5E1

## Abstract

Hydrogen sulfide (H_2_S) can be synthesized in mammalian cells by cystathionine *γ*-lyase (CSE) and/or cystathionine *β*-synthase (CBS). Both CSE and CBS are expressed in rat gastric tissues but their role in human gastric neoplasia has been unclear. The aims of the present study were to detect CSE and CBS proteins in human gastric cancer and determine the effect of exogenous NaHS on the proliferation of gastric cancer cells. We found that both CSE and CBS proteins were expressed in human gastric cancer cells and upregulated in human gastric carcinoma mucosa compared with those in noncancerous gastric samples. NaHS induced apoptosis of gastric cancer cells by regulating apoptosis related proteins. Also, NaHS inhibited cancer cell migration and invasion. An antigastric cancer role of H_2_S is thus indicated.

## 1. Introduction

Hydrogen sulfide (H_2_S) has long been considered as a toxic gas with smell of rotten eggs. H_2_S can be generated endogenously in the mammalian tissues [1, 2]. H_2_S can be produced in mammalian cells from sulfur-containing L-cysteine with the catalyzation by pyridoxal-5′-phosphate-dependent enzymes-cystathionine *γ*-lyase (CSE) and/or cystathionine *β*-synthase (CBS) [1, 3]. H_2_S has been recognized as one of the four gasotransmitters together with NO, CO, and ammonium [2], and it plays an important regulatory role in numerous physiological or pathophysiological processes in our body. CSE is dominantly expressed in the cardiovascular system and H_2_S functions as a vasodilator and cardiac protector [1, 4–8]. In contrast, CBS is mainly expressed in nervous system [1, 2]. In recent years it has been found that gastric tissues express both CSE and CBS [9]. The anti-inflammatory protective effect of H_2_S on the integrity of gastric mucosa has been investigated [10], but the role of H_2_S in gastric neoplasia and the underlying mechanisms are unknown. To this end, we studied expression of CSE and CBS in human gastric cancer samples and explored the effects of exogenous H_2_S donor NaHS on the phenotypic functions of gastric cancer SGC7901 cells.

## 2. Materials and Methods

### 2.1. Gastric Tissue Samples

Ten human gastric carcinoma samples were obtained from patients during gastrectomy surgery (9 males and 1 female aged from 50 to 72). Cancer samples were taken from the center of tumor and noncancerous samples were obtained 5–7 cm away from tumors. All samples were verified by pathology tests. All work was conducted in accordance with the Declaration of Helsinki and Ethical approval was given by the Ethics Committee of Harbin Medical University (number 2008-8).

### 2.2. Cell Culture and Reagents

SGC 7901 cells were purchased from the Cell Bank of the Institute of Life Science, Chinese Academy of Sciences, Shanghai, China. Cells were cultured in Dulbecco's modified Eagle medium (DMEM, HyClone) supplemented with 10% fetal bovine serum (FBS) (HyClone). NaHS, DL-propargylglycine (PPG), and hydroxylamine (HA) were from Sigma (St. Louis, MO, USA). Anti-CSE and anti-CBS monoclonal antibodies were from Abnova (Taiwan). The antibodies for Bax, cytochrome C, caspase 3, cyclin D1, p21, p27, and actin were purchased from Santa Cruz Biotechnology (Santa Cruz, CA, USA). Anti-MMP-2 and anti-MMP-9 antibodies were from Thermo Scientific (Waltham, USA).

### 2.3. Cell Viability Assay

Cell viability was determined by measuring the 3-(4,5-dimethylthiazol-2-yl)-2,5-diphenyltetrazolium bromide (MTT) dye absorbance of viable cells. Briefly, cells were seeded in 96-well microtiter plates with 4,000 cells per well. After growing for 24 hours, cells were exposed to the indicated concentrations of NaHS, PPG, and HA for another 24 hours. Cells of each well then were incubated with 20 *μ*L MTT for 4 hours at 37°C. Formazan crystal formed in viable cells was solubilized by adding 150 *μ*L DMSO. Absorbance at 490 nm was measured on ELx800 microtiter plate reader (BioTek).

### 2.4. Fluorescence Microscopy

For apoptosis assay, 5 × 10^4^ cells per well were plated on 24-well plates and cultured for 24 hours before the indicated concentrations of NaHS, PPG, and HA were added and cultured for 24 hours. Cells were washed and exposed to 10 mg/L Hoechst 33258 at room temperature in the dark for 10 min and 10 mg/L propidium iodide (PI) for 10 min. The cells were observed under a fluorescence microscope (Olympus IX71).

### 2.5. RT-PCR

SGC7901 cells were washed with ice cold phosphate-buffered saline (PBS) and scraped using a rubber policeman and collected into an Eppendorf tube. One mL of Trizol (Invitrogen) was added and total RNA was extracted according to manufacturer's instructions. RNA was treated with RNase-free DNase (Promega) and extracted again. 1 *μ*g of RNA was reverse-transcribed into the first strand cDNA, and PCR was run under the condition 1 cycle of 94°C for 2 min, 32 cycles of 94°C for 30 seconds, 55°C for 30 seconds, and 72°C for 1 min and then 1 cycle of 72°C for 4 min. Primers designed for the PCR were as follows: CSE (forward primer: 5′-GTTTCTTGCAAAACTCTCTTGG-3′ and reverse primer: 5′-TTCTTGAGGAAAATCTCAGCAT-3′); CBS (forward primer: 5′-TCTCACATCCTAGACCAGTACC-3′ and reverse primer: 5′-CTTGTCCACCACCGTCCTGTCC-3′). RT minus reactions also were included.

### 2.6. Western Blotting

Human gastric tissues were snap-frozen in liquid nitrogen after gastrectomy and stored under −80°C. Before being lysed, 100 mg of tissue samples was ground in liquid nitrogen in a mortar and powders were poured with liquid nitrogen into an Eppendorf tube, and 100 *μ*L of lysis buffer was added and put on ice for half an hour. For SGC7901 cells, cells from a 25 cm^2^ flask were washed twice in ice cold PBS and lysed in 100 *μ*L RIPA lysis buffer (50 mM Tris (pH 7.4), 150 mM NaCl, 1% NP-40, 0.5% sodium deoxycholate, 0.1% SDS and 2 mM sodium orthovanadate, and 1 mM EDTA and 1 mM phenylmethylsulfonyl fluoride) on ice. Cell lysates were then centrifuged at 14,000 g for 20 min at 4°C. The supernatant was recovered and protein concentration was detected by Bradford method. Proteins of 20–40 *μ*g were resolved by SDS-polyacrylamide gel electrophoresis (SDS-PAGE) and then transferred to a nitrocellulose membrane (Pall Corp.). The membrane then was immunoblotted with the indicated primary antibodies and detected by using horseradish peroxidase-conjugated secondary antibody and enhanced chemiluminescence (Pierce). Densitometric analysis was performed by Quantity One Analysis Software (Bio-Rad).

### 2.7. Cell Migration Assay

In this assay, 2 × 10^5^ cells/mL were seeded in 12-well plates and cultured to confluence. A 200 *μ*L pipette tip was used to make a straight scratch. For the control group, DMEM containing 0.1% serum was used, whereas, for H_2_S group, NaHS was added in addition to DMEM and 0.1% serum. After 12 h, migration distance of cells was calculated.

### 2.8. Cell Invasion Assay

Invasion assay was carried out in 12-well plate with hanging PET inserts (pore size, 8 *μ*m) (Millipore). The PET membranes were coated with Matrigel (Sigma). In the upper compartment, 400 *μ*L of cells (1.5 × 10^5^/mL) were seeded with DMEM containing 0.1% serum. To the lower compartment, 1.5 mL of DMEM containing 10% fetal bovine serum was added as a chemoattractant. After 20 h of incubation, cells on the upper side of the inserts were removed. Then cells that transmigrated through the Matrigel and membrane were fixed and stained with 0.1% crystal violet. Cell numbers were counted in ten randomly selected fields under a light microscope with 200 time magnification.

### 2.9. Statistical Analysis

All data were expressed as mean ± SEM. ANOVA was used to compare values of test and control samples. A value of *P* < 0.05 was considered statistically significant.

## 3. Results

### 3.1. Upregulation of CSE and CBS Protein Expression in Gastric Carcinoma

**Figure 1:**
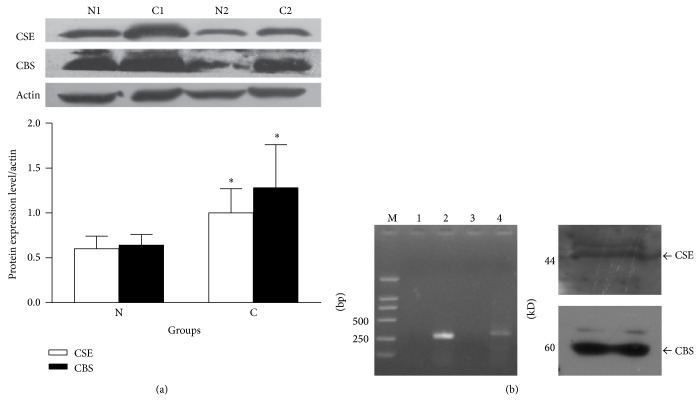
Upregulation of CSE and CBS expression in gastric carcinoma. (a) Tissue lysates from the gastric carcinoma and adjacent noncancerous tissue were immunoblotted with anti-CSE or anti-CBS antibodies. The 2 representative pairs of samples were shown. C, gastric carcinoma; N, adjacent noncancerous tissue. The right panel indicates the quantitative representation. *n* = 10, ^*^ *P* < 0.05 versus corresponding N group. (b) The expression of CSE and CBS in gastric cancer SGC 7901 cells. The left panel shows CSE and CBS mRNA expression in SGC7901 cells. Expected CSE and CBS RT-PCR products are 282 bp and 317 bp, respectively. M: DL2,000 DNA marker; 1: no reverse transcription control for CSE; 2: CSE RT-PCR; 3: no reverse transcription control for CBS; 4: CBS RT-PCR. The right panel shows CSE or CBS protein expression in SGC7901 cells. Protein sizes were 44 kDa and 60 kDa, respectively.

To investigate the role of H_2_S in gastric tumorigenesis, we analyzed the expression of CSE and CBS in 10 gastric primary tumors with the adjacent nontumor gastric tissues. We found that the expression of both CSE and CBS proteins was significantly higher in gastric carcinomas than in adjacent noncancerous gastric tissues (*n* = 10, *P* < 0.05) (Figure 1). We then detected CSE and CBS expression in human gastric cancer cells, SGC 7901 cell line. As shown in Figure 1(b), RT-PCR displayed 282 bp of expected CSE PCR product and 317 bp CBS product. Western blotting revealed major bands for CSE and CBS proteins. Both CSE and CBS were expressed at transcription and protein levels.

### 3.2. H_2_S Reduced Cell Viability of SGC 7901 Gastric Cancer Cells

**Figure 2:**
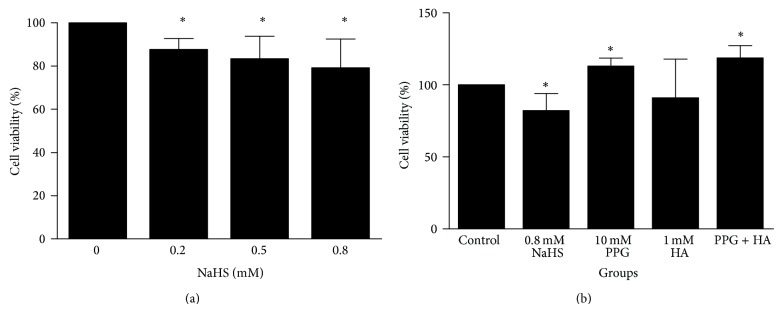
The effect of NaHS on SGC7901 cell viability. (a) NaHS significantly reduces cell viability at the concentrations of 0.2, 0.5, and 0.8 mM. The cells were treated with NaHS for 24 h. (b) Effects of PPG and HA on cell viability. Data were obtained from three independent experiments. ^*^ *P* < 0.05 versus control. *n* = 3.

To assess the effect of H_2_S on cell viability of cloned gastric cancer cells, we exposed these cells to the indicated concentrations of NaHS. When cells seeded at low density to the plates, NaHS treatment significantly increased cell death, compared with the control, in a concentration-dependent manner at concentrations from 0.2 to 0.8 mM (Figure 2). Cell viability was enhanced by PPG alone, but not by HA alone (Figure 2).

### 3.3. H_2_S Induces Apoptosis of SGC 7901 Gastric Cancer Cells

**Figure 3:**
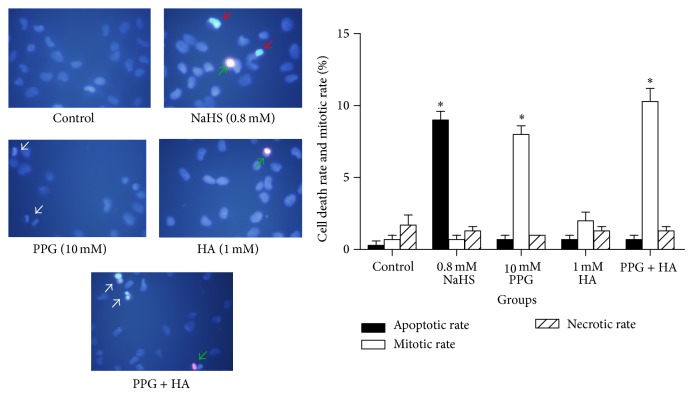
NaHS induced apoptosis of gastric cancer. Apoptosis of gastric cancer cells was determined by Hoechst and propidium iodide staining. Red arrow indicates apoptotic cell nuclei; white arrow is used to indicate mitotic nuclei and green arrow to necrotic nuclei. ^*^ *P* < 0.05 versus control. *n* = 3.

**Figure 4:**
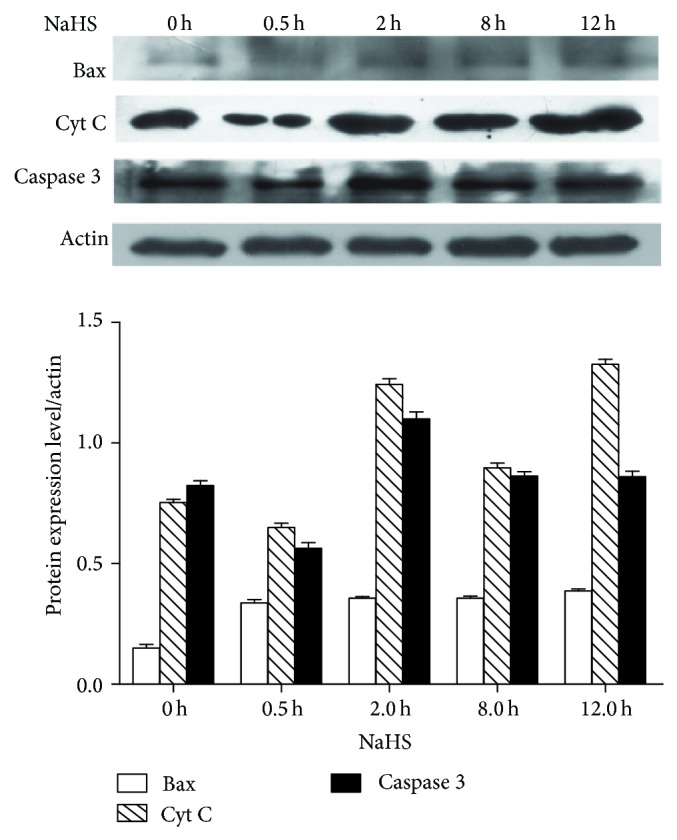
NaHS increased the levels of Bax, caspase 3, and Cyt C in SGC7901cells, detected by western blotting.

**Figure 5:**
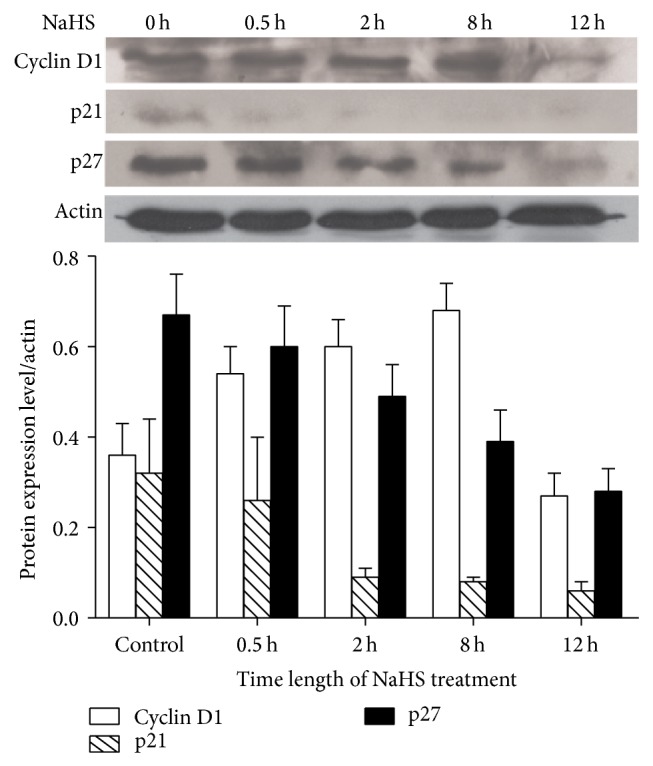
The effect of NaHS on the expression of cell cycle proteins, cyclin D1, p21, and p27 by western blotting. Cyclin D1 was upregulated, but p21 and p27 were downregulated by 0.8 mM NaHS incubation.

To investigate whether H_2_S is involved in apoptosis, we performed apoptosis test using Hoechst-Propidium Iodide staining of cells with different treatments. As shown in Figure 3, NaHS treatment enhanced apoptotic rate of cells. PPG increased mitotic rate. The levels of apoptosis-related proteins, Bax, Cyt C, and caspase 3 were increased after NaHS treatment (Figure 4). We next sought to reveal the role of NaHS on the expression of cell cycle proteins. Cyclin D1 was upregulated during 0.5 h, 2 h, and 8 h, but downregulated at 12 h of NaHS treatment. On the other hand, cell cycle inhibitors p21^waf1/cip1^ and p27^kip1^ were downregulated by NaHS in a time-dependent manner (Figure 5).

### 3.4. NaHS Inhibited Gastric Cancer Cell Migration and Invasion

**Figure 6:**
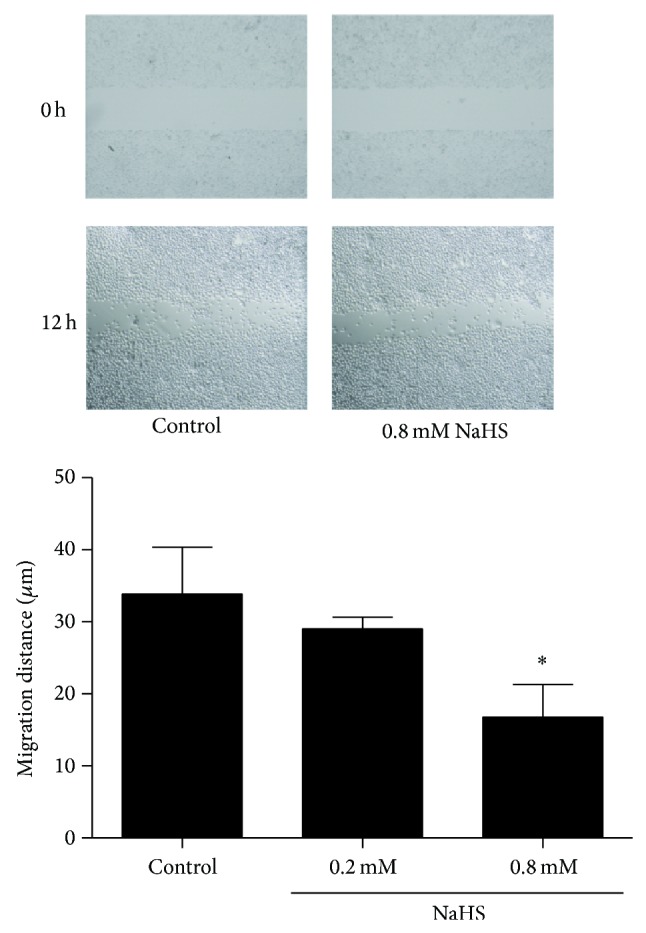
NaHS reduces cancer cell migration. Gastric cancer cells, SGC7901, were cultured in the absence or presence of NaHS. The effects of NaHS on cell migration were determined by a scratch assay. ^*^ *P* < 0.05 versus control. *n* = 3.

**Figure 7:**
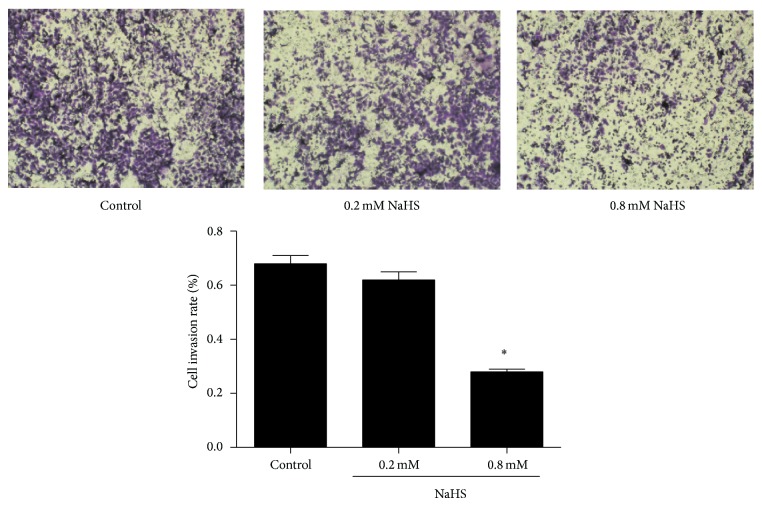
NaHS inhibits cancer cell invasion. Cancer cell invasion was performed in Boyden Chambers with hanging inserts. Cells transmigrated through the matrix gel were calculated based on the cells seeded on the upper chambers. NaHS was added to the upper chambers and cell invasion ratio was determined. ^*^ *P* < 0.05 versus control. *n* = 3.

**Figure 8:**
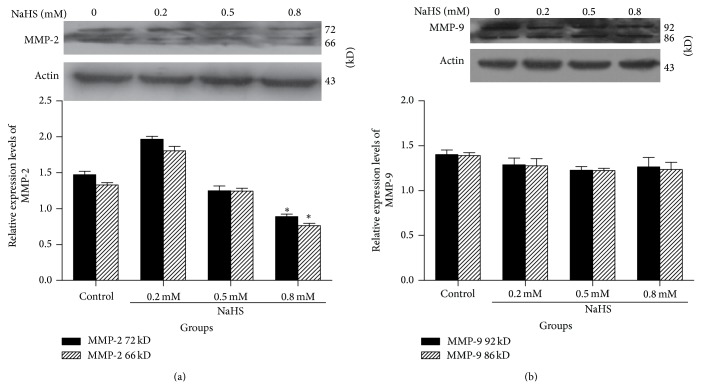
The protein expression levels of MMP-2 and MMP-9 during NaHS treatment. Cell lysate of SGC7901 treated with NaHS was immunoblotted with MMP-2 or MMP-2 antibody and protein expression level of both proteins was determined. (a) MMP-2 expression and (b) MMP-9 expression were determined. ^*^ *P* < 0.05 versus control. *n* = 3.

We further examined the effect of NaHS on SGC7901 cell migration. As shown in Figure 6, 0.8 mM NaHS significantly reduced cell migration in a scratch assay. NaHS-induced delay of coverage of the scratched area by cell migration is unlikely due to the reduced cell proliferation because the assay was carried out in presence of 0.1% serum to essentially stop cell proliferation. To evaluate the contribution of H_2_S on cell invasion, we added NaHS to the upper inserts of Boyden Chambers. As shown in Figure 7, 0.8 mM NaHS inhibited cancer cell invasion. To further determine the mechanisms of involvement in cell invasion, we tested MMP-2 and MMP-9 expression during NaHS treatment. As shown in Figure 8, 0.8 mM NaHS significantly attenuated MMP-2 expression, but there was no significant effect of NaHS observed on MMP-9 level.

## 4. Discussion

The effects of H_2_S on the cardiovascular system [7, 11–14] and the liver [3] have been intensively investigated in recent years. The involvement of H_2_S in the regulation of physiological gastric functions has also been explored [9, 10]. But its role in gastric malignancy has been unknown. Because H_2_S regulates cell growth, proliferation [15], and apoptosis [15–17], we speculated that this gasotransmitter may be involved in gastric hyperplasia.

To test our hypothesis, we firstly collected human gastric cancer tissue samples and performed western blotting to determine the expression levels of H_2_S-producing enzymes CSE and CBS. Our result showed that CSE and CBS were both expressed in noncancerous gastric tissues. Interestingly, both CSE and CBS were upregulated in gastric carcinoma mucosa. This indicates that the production of endogenous H_2_S is elevated in cancerous tissues as a compensatory mechanism against the cancer development. Alternatively, this upregulation may promote tumor growth as a pathogenic mechanism. Our pharmacological intervention study with NaHS, an exogenous H_2_S donor, on cultured SGC7901 cells helps to exclude the tumor-promoting role of H_2_S since NaHS treatment inhibits cancer cell proliferation, migration, and invasion. NaHS has a fast releasing rate in aqueous solution and produces one-third of free H_2_S at neutral pH. NaHS decreased cancer cell viability at concentrations as low as 200 *μ*M (Figure 2). This concentration of NaHS will give 60–70 *μ*M free H_2_S. Although this concentration is still higher than the estimated physiological range of H_2_S in the circulation at low micromolar to high nanomolar levels, it is certainly perceivable as a therapeutic concentration. Moreover, the concentration of H_2_S on the epithelial surface of the stomach mucosa can reach 0.5 mM, because H_2_S generated by CSE and CBS in gastric mucosa can be retained by the gastric adherent mucus gel layer [18].

We also determined the role of endogenous H_2_S in cancer cell viability by testing the effects of CSE inhibitor PPG and CBS inhibitor HA on the viability of SGC7901 cells. PPG alone, but not HA alone, increased cell viability (Figure 2). Furthermore, PPG increased mitotic rate of SGC7901 cells, but HA alone failed to do so (Figure 3). These results indicate that CSE, rather than CBS, plays a major role in gastric production of H_2_S [9] and gastric cancer development. Future determination of endogenous H_2_S levels in normal gastric tissues and gastric cancer tissues will strengthen this notion. Our previous studies have shown that H_2_S was endogenously produced in a colon cancer cell line (WiDr) and colon tissues through the activities of both CSE and CBS. Butyrate and NaHS decreased cell viability in a dose-dependent manner. However, silence of CBS, not CSE, decreased butyrate-stimulated H_2_S production and reversed butyrate-inhibited cell viability [19]. It appears that CSE and CBS play different roles in endogenous H_2_S production along gastrointestinal tract.

The proapoptotic effect of NaHS on SGC7901 cells is demonstrated in this study. This effect may be mediated by NaHS-induced upregulation of Bax, Cyt C, and caspase 3. The activation of intrinsic pathway during apoptosis is triggered by Bax translocation to mitochondria, followed by cytochrome C release from mitochondria and activation of caspase 3. We also assessed the changes in cell cycle control protein levels, including oncogene cyclin D1 and tumor suppressor genes p21^waf1/cip1^ and p27^kip1^ expression, with NaHS treatment. The expression of cyclin D1 protein was increased with 8 h of incubation with NaHS, but decreased at 12 h of NaHS incubation. This expression pattern of cyclin D1 was accompanied by the decreased expression of p21^waf1/cip1^ and p27^kip1^ proteins. Cell cycle progression is controlled by cyclins and cyclin-dependent kinase. Cyclin D1 is a major oncogene overexpressed in many types of cancers. Elevated expression of cyclin D1 shortens G1 phase of the cell cycle to facilitate cell cycle progression through G1 checkpoint. Both p21^waf1/cip1^ and p27^kip1^ proteins are cyclin-dependent kinase inhibitors (CDKI). Decreased expression of p21^waf1/cip1^ and p27^kip1^ proteins of gastric carcinoma cells by NaHS treatment suggests that more cancer cells may proceed through the G1 checkpoint to S and G2 phases.

To determine the effect of H_2_S on cancer cell migration and invasion, we carried out the “scratch” assay on cultured confluent SGC7901 cells. Our finding showed that NaHS inhibited cancer cell migration and invasion. For cell invasion to occur, extracellular matrix must be degraded. MMPs are potent proteinases for cancer cells to degrade the matric gel. MMP-2 and MMP-9 are two major matrix metalloproteinases [20]. Our immunoblotting tests suggested that the inhibitory effects of H_2_S on cell invasion might be through the downregulation of MMP-2, not MMP-9. H_2_S inhibits cell migration (the initial step for cell invasion) and MMP-2 expression (critical step for cell invasion) and, therefore, blocks gastric cancer cell invasion.

In conclusion, endogenous hydrogen sulfide may play an anticancer role in gastric malignance development by regulating multiple steps. Our in vitro cell culture study shows the potential of a H_2_S donor in restricting the growth and migration of gastric cancer cells. These observations should be extended to whole animal in vivo studies, such as using gastric cancer-implanted or gastric cancer-metastasis animal models, before the therapeutic application of H_2_S donors against gastric cancer development can be realized.
